# AGEISM IN HEALTH CARE: 72 IS NOT A DIAGNOSIS

**DOI:** 10.1093/geroni/igz038.307

**Published:** 2019-11-08

**Authors:** Phyllis A Greenberg, Tarynn Johnson

**Affiliations:** St. Cloud State University, St. Cloud, Minnesota, United States

## Abstract

This poster examines what value, if any, there is in using age as a predictor or impetus for testing, examining and diagnosing older adults. In a cross sectional survey (Davis et al. (2011) used the Expectations Regarding Aging Scale to assess primary care clinicians perceptions of aging in the domains of physical/mental health and cognitive functioning. Sixty-four percent of respondents agreed with the statement “Having more aches and pains is an accepted part of aging while 61% agreed that the “Human body is like a car when it gets old it gets worn out. And 51% agreed that one should expect to become more forgetful with age while 17% agreed that mental slowness is impossible to escape. How might these attitudes and biases effect how older adults are diagnosed, heard, spoken to, and treated (medical treatment as well as patient/professional interaction)? Are older patients/clients underserved or over served? Is forgetting where you put your keys always or even usually a sign of dementia? How helpful then is the use of age and are there other factors that should and can take precedence? What do we know and what don’t we know if we know someone’s age? Successful and innovative tools are explored that acknowledge age biases and strategies are presented to change age biases in education, training and practice.

